# Parapharyngeal abscess in children - five year retrospective study

**DOI:** 10.1016/S1808-8694(15)30544-9

**Published:** 2015-10-19

**Authors:** Pedro Miguel dos Santos Marques, Jorge Eduardo Freitas Spratley, Laurentino Manuel Mendes Leal, Eduardo Cardoso, Margarida Santos

**Affiliations:** 1 ENT Resident - Hospital de S.João E.P.E. – ENT Volunteer Assistant –Porto University Medical School - Portugal; 2 Attending Physician – ENT Hospital de S.João E.P.E. – Adjunct Professor of ENT – Porto University Medical School - Portugal; 3 ENT Resident - Hospital de S.João E.P.E. – ENT Voluntary Assistant – Porto University Medical School - Portugal; 4 Head of the ENT Service - Hospital de S.João E.P.E. – ENT Guest Physician – Porto University Medical School - Portugal; 5 ENT Director - Hospital de S.João E.P.E. – Porto Portugal. Hospital de S.João- E.P.E. Porto Portugal

**Keywords:** abscess, pharynx, neck

## Abstract

Lateropharyngeal and retropharyngeal abscesses are potentially life threatening infections in children

**Aim:**

To review the etiologic, clinical, and imaging signs of lateropharyngeal and retropharyngeal abscesses in children as well as treatment-outcomes and complications using a surgical trans-oral approach.

**Method:**

Retrospective analysis of 11 children, hospitalized in the last 5 years, with a diagnosis of lateropharyngeal (n = 8) and retropharyngeal (n = 3) abscesses, ages ranging from 0 to 12 years old. Charts and CT scans were reviewed.

**Result:**

The average age of presentation was 3.3 years. Neck stiffness (64%) and odynophagia (55%) were the most common symptoms. Fever (64%), stiff neck (64%), bulging of the oropharyngeal wall (55%), mass in the neck (55%) and lymphadenopathy (36%) were the most prevalent physical findings. All these patients were submitted to surgical drainage using a trans-oral approach in the first 48 hours after admission. About 82% of the patients showed improvement after 48 hours, and 100% after 72 hours, without any complications.

**Conclusion:**

Based on the good clinical outcomes and low incidence of complications, the present study suggests that antibiotic therapy complemented with a timely surgical treatment, is a valid treatment option in refractory parapharyngeal abscesses.

## INTRODUCTION

Deep neck infections and subsequent formation of abscesses in the fascial planes adjacent to the pharynx still happen despite the advent of antibiotics. Their occurrence may be associated with significant rates of morbidity and mortality, mostly due to their multiplicity of complications, which includes obstruction of the airway, rupture of the abscess into the pharynx or trachea, empyema, mediastinitis, erosion of the carotid artery, jugular thrombophlebitis or thrombosis of the cavernous sinus.^1^, ^2^, ^3^ Diagnosis of these potentially life-threatening infections is difficult when they appear in a child, mainly because complaints and clinical signs are less clear and physical examination is more difficult to perform. Nevertheless, lateropharyngeal (LP) and retropharyngeal (RP) abscesses require prompt diagnosis and early management which frequently involves surgical drainage to achieve the best results. However, the appropriate timing to undergo a surgical procedure is still controversial. The present study reviews recent experience in the management of pediatric LP and RP abscesses at our Department, with a special emphasis on the early surgical approach used in the treatment of these children.

## MATERIALS AND METHODS

The clinical records of all children consecutively admitted at the department of otorhinolaryngology of a terciary care university hospital with a diagnosis of LP/RP abscesses between May 2001 and April 2006 were retrospectively reviewed. Peritonsillar abscesses were excluded. Factors such as sex, age, possible aetiology, clinical symptoms, physical findings, results of blood tests, findings on imaging studies, treatment, clinical outcomes, and complications were analysed.

## RESULTS

Eleven children with an average age of 3.3 years (mínimum 9 months old, maximum 7 years old) were identified. Almost half of the patients were less than two years old, and the group presented a male/female ratio of 4:1. Acute tonsillitis or nasopharyngitis preceded the deep neck infection in 45% and 9% respectively, while in 36% of cases no cause was identified. The most common symptoms included torticollis (64%) and odynophagia (55%) which started on average 3.3 days before admission (Table 1).

**Table 1:** Presenting symptoms at admission

Symptoms	Number of patients	%
Immobility of neck	7	64
Odynophagia	6	55
Refusal of oral intake	5	45
Trismus	4	36
Lethargy	3	27
Drooling	1	9
Headache	1	9
Disorder of the voice	1	9
Nasal discharge	1	9

**Figure 1:**
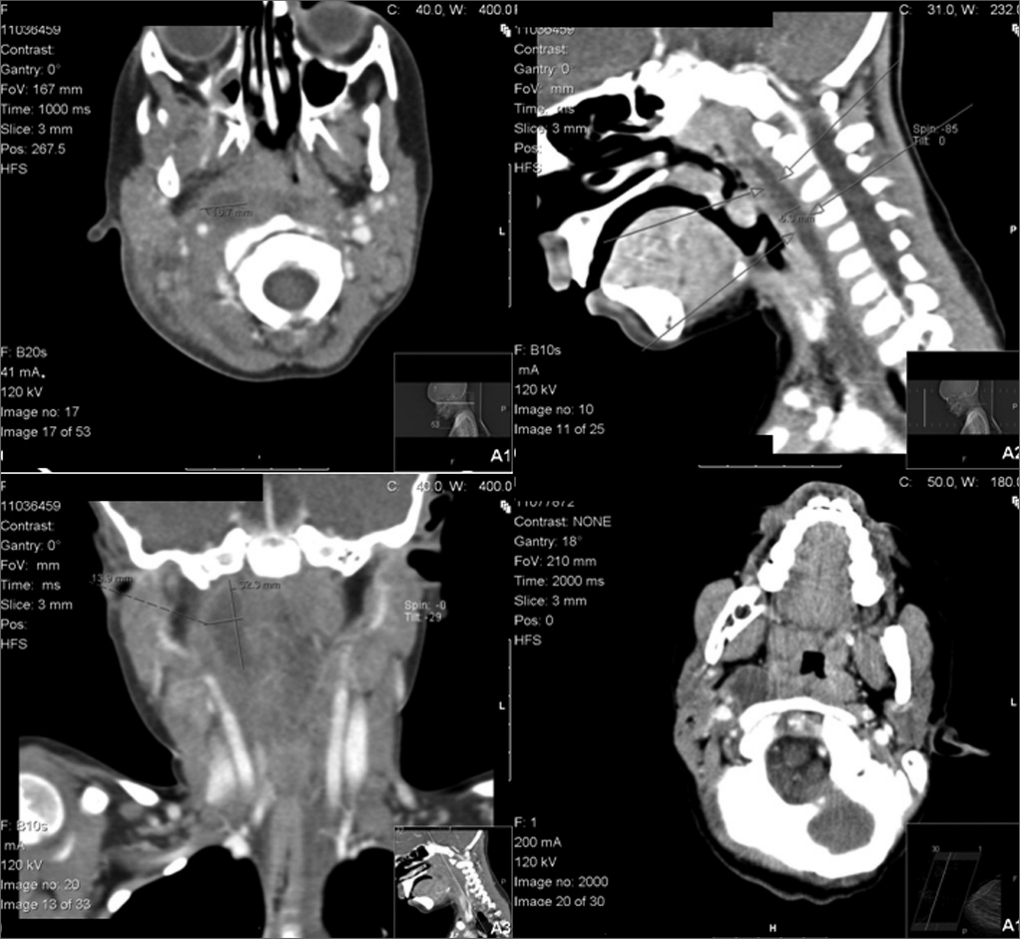
A. Latero and Retropharyngeal abscess (CT scan, axial view). B. Latero and Retropharyngeal abscess (CT scan, sagital view). C. Lateropharygeal abscess (CT scan, coronal view). D. Lateropharyngeal abscess (CT scan, axial view)

On physical examination the most prevalent findings were fever (64%), stiffness in the neck (64%), bulging of the oropharyngeal wall (64%), masses in the neck (55%), and lymphadenopathy (36%). All patients presented more than one of these symptoms or physical findings at the time of admission (Table 2). Noticeably, 55% of patients were under antibiotic therapy before admission, namely amoxycilin/clavulanic acid (45%) or claritromycin (18%). The diagnostic workup included, in every child, a computed tomographic (CT) scan with intravenous contrast that showed areas of low-attenuation with peripheral enhancement in eight cases of LP abscesses and in three children with RP abscesses. The size of the abscesses varied from 15 mm to 32 mm on the longest axis, with an average of 22.5 mm (Figure 1). Blood tests revealed increased white blood cell counts (WBC) (average of 20.93* 109/?l) and reactive C protein (RCP) (average of 146 mg/ml) in every patient, while 54% of these patients presented with mild anaemia (average haemoglobin levels of 11.5 g/dl).

**Table 2:** Physical findings at admission

Physical findings	Number of patients	%
Fever	7	64
Stiff neck	7	64
Bulging of oropharyngeal wall	7	64
Mass in the neck	6	55
Lymphadenopathy	4	36
Torticollis	3	27
Facial edema	1	9
Nasal discharge	1	9

Upon admission every patient started intravenous antibiotic therapy with associated administration of amoxycilin/clavulanic acid and amykacin. No patient required to have the airway secured as a result of respiratory distress. All children were surgically managed using a trans-oral approach under general anaesthesia. In 45% of cases the drainage was performed within the first 24 hours and in 55% within the first 25 to 48 hours. These children who were only admitted to the surgery room after 24h, presented, at admission, a less toxic state and aggravated their condition in the first hours of medical treatment. The surgical approach in RP abscesses consisted of a vertical incision medial to the palatopharyngeal arch of the affected side. In LP abscesses, exploration of the lateropharyngeal space was done through the tonsillar fossa after ipsilateral tonsillectomy. Purulent fluid was drained in 73% of the cases. Cultures were positive in 25% of samples, and all yielded group A Streptococcus pyogenes. Blood cultures were positive in only one patient for Staphylococcus epidermidiis and Streptococcus sanguiinis.

Clinical improvement represented by reduction of fever, recovery of stiffness of the neck, increase of oral intake, and reduction of cervical adenopathy or swelling in the neck was observed on the first 24 hours after drainage in 82% of the patients. In one child a second surgical approach was needed. All patients improved clinically in less than 72 hours, and there were no postoperative complications. The length of hospital stay varied between 7 and 17 days with an average of 10.5 days. Every patient was followed for six months, without evidence of recurrence of the pathologic conditions.

## DISCUSSION

Formation of abscess in the fascial planes adjacent to the pharynx is a severe occurrence in children.^1^ Usually both RP and LP abscesses arise, consequent to oropharyngeal infections that spread either by direct continuity or by lymphatic drainage.^1^, ^4^, ^5^ These infections tend to decrease in incidence in older children, apparently not only because of progressive atrophy of the RP lymph nodes but also as a possible result of lesser incidence of upper respiratory infections with increasing age.^6^, ^7^ On the other hand, an immature immune system in younger children may also contribute to an increased risk.

The anatomy of the cervical fascial spaces is a significant variable in determining the presentation, course, and outcome of the infection.^1^ The relevant anatomic structures enclosed in this area (i.e., the carotid sheath, digestive tract, and airway) and continuity of neck spaces within the mediastinum makes this area a “danger zone”. Since this is so, accurate knowledge of the anatomy is essential for the physician to be aware of the possible course that progression of the infection may follow^1^, ^8^, as well as to plan surgical treatment when necessary.

The clinical presentation of abscesses in these spaces is commonly determined by the effects of inflammation on the structures within and bordering them. Differently from adults, who often present with typical signs and symptoms, children tend to have a more subtle presentation, seldom being able to verbalize or cooperate with the physical examination.^4^ According to Coticchia and colleagues^5^, younger children have uncharacteristic presentations of deep neck abscesses that closely mimic signs and symptoms of viral upper respiratory tract infection such as agitation, cough, lethargy, and rhinorrhea, increasing the difficulty of establishing an accurate diagnosis. Keeping in mind the diagnosis of RP and LP abscesses in children with torticollis or stiff neck may lead to earlier diagnosis, timely treatment, and therefore fewer cases of morbidity.^9^ Like previous reports^3^, ^5^, ^7^ the current study showed, as most usual symptoms, fever, limited motion of neck, and odynophagia. Moreover, as expected in an infectious event, a rise in WBC count and RCP was reported in all patients. Also of note in our series was the presence of mild anaemia in more than half of the patients, a fact probably explained by the aggressiveness of this type of infection.

**Figure 2:**
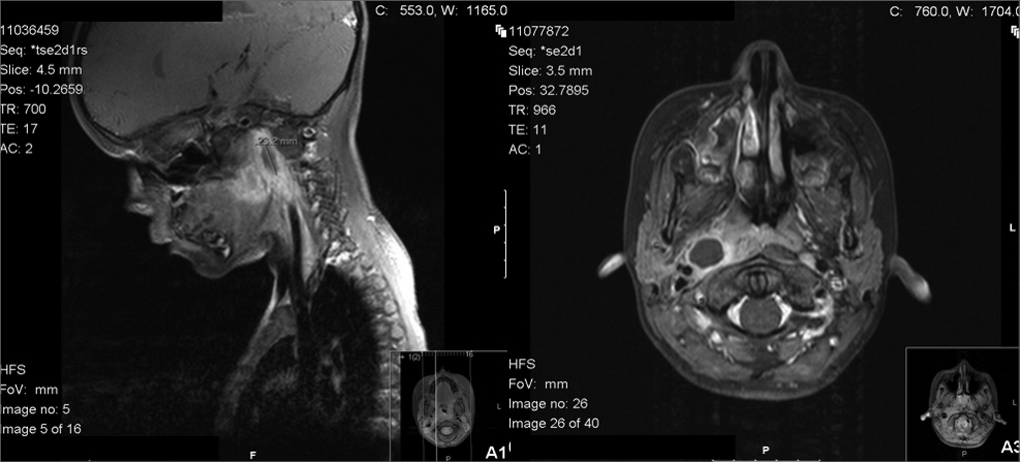
A. Lateropharyngeal abscess (MRI scan, sagittal view). B. Lateropharyngeal abscess (MRI scan, axial view)

The results of cervical ultrasound studies in this group of pediatric patients were frequently non-conclusive, probably due to decreased collaboration by the patients and to the deep localization of the abscesses. In contrast, CT proved very effective in the evaluation of these deep structures, allowing us to identify the spread of the infection beyond what was clinically evident. Furthermore, progression of infection from cellulitis to abscess can be traced, thereby optimizing a timely decision on the surgical procedure and planning of a surgical approach.^10^ Interestingly, analysis of the CT data in our patients showed a predominance of LP infections in comparison with studies by other authors that showed a higher prevalence of RP abscesses.^11^, ^12^, ^13^, ^14^ Magnetic resonance imaging (MRI) may also be used in diagnosis and follow-up of these infections, with the advantage of an absence of radiation inflicted on the child. In this series, all patients underwent MRI as a follow-up imaging control (Figure 2).

Previous investigators reported that abscesses in proximity to the oropharynx, often contain microflora indigenous to this region, while abscesses located distant from the oropharynx tend to grow organisms commensal to the skin.^7^, ^15^ Accordingly, Coticchia and colleagues^5^ found group A streptococci and normal oropharyngeal flora more commonly in RP and LP than in submandibular or submental abscesses in which Staphylococcus aureus predominates. In our series 25% of cultures yielded isolates of group A Streptococcus pyogenes.

The cornerstone of management consists in antimicrobial therapy, intravenous hydration, control of pain and the airway, and surgical drainage.^1^ In another series, Huang and his team reported a 10% rate of tracheostomies in patients with deep neck abscesses.^16^

Lately, the surgical treatment of deep neck abscesses has been the subject of controversy in medical publications. Some clinicians recommend early surgical drainage in most or all cases^16^, ^17^, ^18^, ^19^, ^20^, while others are strongly in favour of a more conservative non-surgical initial treatment.^3^, ^13^,^21^, ^22^, ^23^, ^24^, ^25^

Studies evaluating management without surgical drainage showed variable results, ranging from a high rate of resolution of 75%^25^ to a high rate of failure with only 31.5% of patients recovering on medical therapy alone.^23^ Both series pertained to patients with RP abscesses only and included a significant number of patients, 68 and 54 respectively. However these authors advise close monitoring of the patient and consideration of surgical intervention in those whose clinical condition does not improve on medical therapy.^1^ Furthermore, other reports claim that use of closed drainage by aspiration^26^, ^27^ produces rates of resolution around 80%. However, these studies reported on small series and did not specify the type of abscesses.

Interestingly, Sichel and colleagues^24^ proposed a distinction between two cervical spaces (anterior or posterior parapharyngeal spaces) that could influence decision-making in relation to timing of the surgical approach. These spaces are separated by the styloid muscles (stylohyoid, stylopharyngeus, and styloglossus muscles), so infection developing in the anterior space showed a more aggressive course and extension to other fascial planes, requiring an early surgical approach; in contrast to the posterior type of infection that evolved in a more benign fashion with fewer complications.

In the present series of patients, the choice was towards early surgical drainage. This approach was associated with good clinical outcomes and an absence of complications or mortality. However the otorhinolaryngologist must take into account the specificity of the regional anatomy and general anaesthesia, especially in patients of pediatric age, whenever opting for surgery. Taking into account the scientific knowledge developed so far, a procedure for deciding the right time for surgery is still debatable, in spite of this small series revealing good outcomes with an early surgical approach. Therefore, appears to be unquestionable that additional studies in this field of research are urgently needed.

## CONCLUSIONS

RP and LP abscesses are clinical entities with a relevant incidence in the pediatric population. They present a diagnostic challenge requiring a high index of suspicion from the physician and in which imaging studies, in particular computed tomography, are essential for diagnosis and surgical planning. Based on the good clinical outcomes and low incidence of complications, the present study suggests that antibiotic therapy complemented with a timely surgical treatment, is a valid treatment option in refractory parapharyngeal abscesses. Nevertheless more research in this topic is desirable.
